# Automatic parcellation of resting-state cortical dynamics by iterative community detection and similarity measurements

**DOI:** 10.3934/Neuroscience.2021028

**Published:** 2021-09-10

**Authors:** Tien-Wen Lee, Gerald Tramontano

**Affiliations:** 1 The Neuro Cognitive Institute (NCI) Clinical Research Foundation, NJ 07856, US; 2 Department of Psychiatry, Dajia Lee's General Hospital, Lee's Medical Corporation, Taichung 43748, Taiwan

**Keywords:** cortex, functional connectivity, functional magnetic resonance imaging (fMRI), functional parcellation, resting state fMRI (rs-fMRI)

## Abstract

To investigate the properties of a large-scale brain network, it is a common practice to reduce the dimension of resting state functional magnetic resonance imaging (rs-fMRI) data to tens to hundreds of nodes. This study presents an analytic streamline that incorporates modular analysis and similarity measurements (MOSI) to fulfill functional parcellation (FP) of the cortex. MOSI is carried out by iteratively dividing a module into sub-modules (via the Louvain community detection method) and unifying similar neighboring sub-modules into a new module (adjacent sub-modules with a similarity index <0.05) until the brain modular structures of successive runs become constant. By adjusting the gamma value, a parameter in the Louvain algorithm, MOSI may segment the cortex with different resolutions. rs-fMRI scans of 33 healthy subjects were selected from the dataset of the Rockland sample. MOSI was applied to the rs-fMRI data after standardized pre-processing steps. The results indicate that the parcellated modules by MOSI are more homogeneous in content. After reducing the grouped voxels to representative neural nodes, the network structures were explored. The resultant network components were comparable with previous reports. The validity of MOSI in achieving data reduction has been confirmed. MOSI may provide a novel starting point for further investigation of the network properties of rs-fMRI data. Potential applications of MOSI are discussed.

## 1. Introduction

Functional brain imaging has become an important resource for understanding brain function and neuropsychiatric conditions. Each neuroimaging tool has its own strengths and limitations, and it is generally agreed that different modalities may contribute complimentary information, such as electrophysiology vs. blood-oxygen-level dependent (BOLD) signals and structural connectivity vs. functional connectivity (FC) [1]. In contrast to the low spatial resolution of electroencephalography, functional magnetic resonance imaging (fMRI) may provide neuro-informatics at higher anatomical precision from tens to hundreds of thousands of voxels. An innate difficulty associated with fMRI lies in how to utilize the high dimensional data to draw conclusions.

It is well known that the temporal dynamics between neighboring fMRI voxels are not totally independent of each other, justifying an application of a reduction strategy to simplify the data structure. In activation designs (i.e., the subjects are challenged with psychological tasks), both parametric (random field theory) and non-parametric (simulation) approaches have been developed to quantify “resolution elements” that may help to eschew stringent Bonferroni correction and hence to provide appropriate statistical inferences [2],[3]. However, in resting state fMRI (rs-fMRI) designs, researchers still heavily rely on predefined regions of interest (ROIs) that are informed by anatomical atlases, not the BOLD data itself, to achieve data reduction (i.e., structural parcellation) [4]–[9]. The resolution of rs-fMRI data can therefore be reduced to tens or hundreds of units. Although widely applied, its validity has been questioned because the correspondence between functionally specialized brain regions and brain areas delineated by anatomical landmarks and cytoarchitectonic structures may be limited [4],[5],[10]–[13]. In other words, the ROIs derived from structural atlases are not necessarily functionally homogeneous, which may result in biased and even faulty network property estimation. Empirical evidence arguing both for and against the aforementioned approach has been reported [4],[5],[7]–[9],[12],[13]. A recent study suggested that despite the flawed biological plausibility, whether an atlas-informed partition of the cortex is a valid data-reduction strategy or not is actually a context-dependent issue [6]. Either taking its validity for granted or rejecting its usefulness blatantly is an arbitrary assertion.

Given the ambiguity and debate described above, it is desirable to parcellate the cerebral cortex based on the similarity of regional and neighboring BOLD dynamics itself to achieve data reduction (i.e., functional parcellation [FP]), instead of resorting to the structural parcellation via pre-defined ROIs. However, this challenging issue in rs-fMRI research is more complicated than it appears. The cortex is organized in distributed hierarchical and parallel manners with intense forward, backward and lateral projections [14]–[16], which may endorse diverse functional specialization of brain regions and sub-regions [17]–[27]. The above observation indicates that FP of the cortex can actually be performed at multiple resolutions that are dependent on the perspectives/scales that researchers wish to explore [28],[29]. However, contradictory evidence suggests that the cortical modules in rs-fMRI research seem to be stable and independent of different methodologies [4],[30]. Although several elegant algorithms have been developed to perform automatic whole-brain FP, a consensus has not been reached yet [31]–[35]. Previous research sampled the cortex uniformly to reduce the data dimensions [28]. Cohen et al. utilized information of the transition of FC maps to delineate the boundaries between functional areas, while Wig et al. applied snowball sampling to identify the neighbors and neighbors of neighbors and so forth to achieve FP [31],[34]. Although the above exemplified methods are persuasive in formulation and comparable in results, they provide fixed partitions and do not address the nature of local homogeneity that is the fundamental observation and rationale to pursue a data-reduction procedure (note: “local homogeneity” is used to avoid confusion with the term “regional homogeneity” in re-fMRI research, ReHo).

This study proposes a fast, iterative algorithm to perform automatic FP that incorporates modular analysis and similarity measurements (named MOSI), the former splitting a module into sub-modules and the latter uniting similar sub-modules into a bigger module. Modules or communities are groups of nodes within a network that are more densely connected to one another than to other nodes [36],[37]. In this research, the neural nodes are the voxels, and the connectivity strengths (edges) between nodes are defined by FC, i.e., correlation strength. Since the (sub-) modules were derived by the connectivity strengths, the voxels with similar BOLD dynamics (thus higher connectivity strengths) tended to aggregate as a community. The modularity of a partition is a metric between −1 and 1 that indicates the quality of the network partition, i.e., the density of edges inside communities compared to that of edges between communities [37],[38]. After partition, the mean BOLD signal of each (sub-) module is correlated with those of the remaining (sub-) modules to constitute a correlation (FC) map. The adjacent (sub-) modules with similar FC maps (and hence smaller “relative distance”) are grouped together as a new module (note: adjacency is defined as 26 possible connections by any points, lines or faces of a 3-dimensional cube). Iteration of the division and unification processes will be carried out until the derived modular structure is stable. The number of modules and, hence, resolution of a partition can be titrated by gamma values (see Material and Methods for details). Since information processing in the cerebral cortex relies on interactions among the distributed areas, the results of FP are subject to further exploration to disclose the constituent communities, which are compared with known brain networks [17]–[19],[35].

## 2. Materials and methods

### 2.1. Subjects, MRI data acquisition and imaging data preprocessing

For detailed information refer to Lee et al.'s previous paper [39], which is briefly summarized below. Thirty-three right-handed healthy subjects were selected from the dataset of the Rockland sample (Enhanced Nathan Kline Institute; NKI-RS), with a mean age of 26.6 years. The data source used by this study met the criteria of “NKI-RS Neuroimaging Data Release”. The institutional ethics committee approved this project. T1-weighted structural MRI (sMRI) and rs-fMRI were included in the analysis. All MRI images covered the whole brain and were acquired by a 3.0 Tesla Siemens Magnetom Trio Tim system, with 260 whole-brain echoplanar images (EPIs) and TR 2.5 seconds (first 5 images were discarded for equilibrium).

FreeSurfer was applied to the T1-weighted sMRI to segment gray matter and white matter and to parcellate the cortical mantle into 70 (Desikan-Killiany Atlas) ROIs [40],[41]. The Analysis of Functional NeuroImages software package (AFNI) was used to process the rs-fMRI data [3], and the analytic streamline developed by Jo et al. was adopted to prepare the rs-fMRI images [4],[5],[42]. The pre-processing steps of rs-fMRI comprised despike, slice-time correction, realignment (motion corrected), registration to T1 anatomy, spatial smoothing (6 mm), and bandpass filtering to 0.01–0.1 Hz. Several regressors were created and modeled as nuisance variables, comprising twelve movement parameters, tissue-based regressors of white matter (global and local) and ventricles, and third-order polynomials to fit baseline drift [42]. The tissue-based regressors were constructed with the help of the segmentation results obtained from FreeSurfer.

### 2.2. MOSI scheme

MOSI is an iterative process, and each iteration mainly comprises two steps to divide and to merge modules. It is notable that different FC maps were constructed by Pearson's correlation for each step (i.e., regional and global, detailed below). In the division stage, the Louvain community detection algorithm was applied to each module derived from the preceding iteration. The first iteration starts from the ROIs of the cortex delineated from the Desikan-Killiany Atlas. In this stage of MOSI, the “regional” correlation map of each module was generated as an input to the Louvain algorithm for community detection (see next section). Suppose a particular module has N voxels, then the correlation map is a matrix with dimension N × N, with each element being the correlation coefficient (CC) of a voxel-pair within that module.

It is noteworthy that although several community detection algorithms can be applied directly to a huge network—the whole brain in this case—the resultant network patterns are inevitably composed of distant brain regions, which is in accord with the fact that the brain is an intensely interactive system [4],[5],[45]. However, what is more important for automatic FP is to emphasize local (neighboring) “homogeneity” in terms of the BOLD dynamics. Another application of community detection in previous rs-fMRI research is to decompose a brain region into several sub-regions [39],[46],[47]. At this stage, MOSI is similar to the latter. If the voxels of a resultant sub-module form disconnected sub-groups, say 2 sub-groups, this disconnected sub-module is further separated into 2 smaller communities because only neighboring voxels are qualified to be grouped as a module in MOSI. Different resolutions were explored by setting gamma values from 0.65 to 0.95 at an interval of 0.05.

The second stage is to unite similar modules into a larger module. In this unification stage of MOSI, the mean time series of each module was calculated, which was correlated with the remaining modules to generate another correlation map (a vector). This FC vector carried the sampled information of the entire cerebral cortex that was used to define similarity, thus a “global” FC map [6]. The similarity among the FC maps was computed for each pair of neighboring modules, which is the square root of the weighted square sum of the difference between two FC maps (weight reflects the voxel number). Similarity was indicated by the relative distance of two FC maps, which was calculated for each pair of neighboring modules. For a similarity index less than 0.05, neighboring modules were unified to become a new module. In other words, if the mean BOLD signals of two neighboring modules showed a high correlation but their (whole-brain) FC vector looked different, they were regarded as separated and not unified, which was expected to possess higher differentiability. It is notable that the entire cortex information is sampled to define relative distance. A large relative distance means that two brain FC maps show substantial differences.

The program ends when the modular structure is stable. Operationally, stop criteria can be evaluated from two perspectives: the output modular structures for two consecutive cycles are either exactly the same or their normalized mutual information is higher than 0.95, in which a 1 percent difference in modular number was allowed. The mean CCs of each resolution of the MOSI results were summarized (Table 1), and the mean CCs were compared across different resolutions and with those in the original ROIs (Table 2). Each hemisphere was treated separately. SUMA (AFNI associated software) was used to illustrate the MOSI partition results.

There are several technical details worthy of mentioning. First, in the beginning of each iteration step, small/fragmented modules with a voxel number less than 10 were identified and united with neighboring modules that showed the strongest correlation strength of no less than 0.50. Tiny modules inherently had fewer neighbors and were believed to be part of a larger neighboring community because of the innate covariance structure of fMRI data and the applied smoothing procedure [2],[3]. Considering the possible number of combinations between (sub-) modules, the computation time for each iteration increases drastically with modular number. This procedure may reduce the number of modules that are to be treated and hence save computation time. Second, for each iteration of MOSI, a permutation is introduced to the order of the modules before computation. Take modules A, B and C as an example. After partitioning, suppose that A is decomposed into A1 and A2, B into B1 and B2, and C into C1 and C2. A1 shows the highest similarity to B2, while B2 shows the highest similarity to C1. If changing the order does not occur, A1 will always be united with B2, and B2 will have no chance to unite with C1—this could not be the optimal solution. This permutation may facilitate the algorithm to converge. Last, since this algorithm works quite quickly for lower values of parameter gamma (e.g., ≤0.8), if the users wish to perform FP at a higher resolution, it is recommended to run MOSI at a lower gamma value to provide a good initial guess as a starting point. In other words, a finer FP is derived from a coarser FP instead of breaking the original ROIs into many small modules and repeating the splitting-unification cycles for the high gamma condition. In this study, the results of gamma 0.70 are based on the results of gamma 0.65, and the results of gamma 0.75 are based on the results of gamma 0.70 and so forth. This simple strategy may substantially decrease computation time.

### 2.3. Community/module detection and comparison

The Louvain method was employed to partition each module into composite sub-modules through optimization of Newman's modularity metric Q [36]–[38], in which 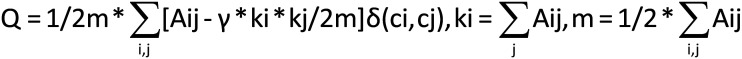, Aij is the connectivity strength between nodes i and j, ci is the community that node i belongs to, δ is the delta function (if ci = cj, delta = 1, otherwise 0), and γ (gamma) is the resolution factor. The Louvain algorithm may accommodate different resolutions by adjusting gamma values and may decompose parametric/weighted networks (in contrast to binary networks in which the connections are either 0 or 1) [43],[44]. A higher gamma value means that the resultant partitions are smaller in size (fewer nodes), and vice versa. The output partitions of MOSI can be compared by normalized mutual information; if two partitions are exactly the same, their mutual information will be one. The above computations of community detection and community comparison were performed using the brain connectivity toolbox (http://www.brain-connectivity-toolbox.net) [37]. The mathematical software Matlab was the platform used to execute the brain connectivity toolbox and was used to manage the MOSI pipeline (Mathworks, Natick, MA, USA).

### 2.4. Exploration of MOSI results

The BOLD time series of the voxels in each MOSI-derived module are expected to be more similar when compared with those in other modules. The validity was examined by an intra-individual analysis in which the mean CC between the voxels within each module was compared with the (averaged) CC between the mean BOLD signals of that module and other modules, with the former expected to be greater than the latter. The null hypothesis was that the CC within each module was the same as the CC between different modules (i.e., the mean difference is zero), and a t-test was performed for each subject.

To assess the influence of resolution, grand mean CCs across seven gamma values for each participant were calculated by taking the mean of CCs from the BOLD dynamics of the voxels of the constituent modules. Similar calculations were applied to the initial partition of the 70 ROIs. Given that Louvain partition is based on the correlation strengths and the neighboring voxels of fMRI tend to be highly correlated, it is inferred that the MOSI-derived modules would show higher intra-modular similarity with finer resolution (i.e., with increasing gamma value, the derived modules shall be smaller in size and with more homogeneous content if MOSI works properly).

The results of MOSI provide a novel starting point to identify networks of functionally coupled regions across the cerebral cortex. The mean representative BOLD dynamics were retrieved to achieve data reduction, and the module was shrunk to a neural node. A new set of FC maps (second level) were constructed accordingly, and the community detection algorithm was applied again to the simplified neuro-informatics to disclose underlying modular structures – large-scale networks. The number of output networks were arbitrarily assigned to 9 by adjusting the parameters of Louvain algorithm. The detailed modular structures derived across 7 gamma values were compared with 5 commonly reported networks in previous rs-fMRI research [28],[48], i.e., visuo-associative, cognitive/attentional fronto-parietal, default-mode, sensorimotor and limbic/paralimbic networks (note: the attentional fronto-parietal network is not a robust finding, which is not always mentioned in previous reports [6],[30],[45]). The results were visualized by AFNI and SUMA.

## 3. Results

**Figure 1.**
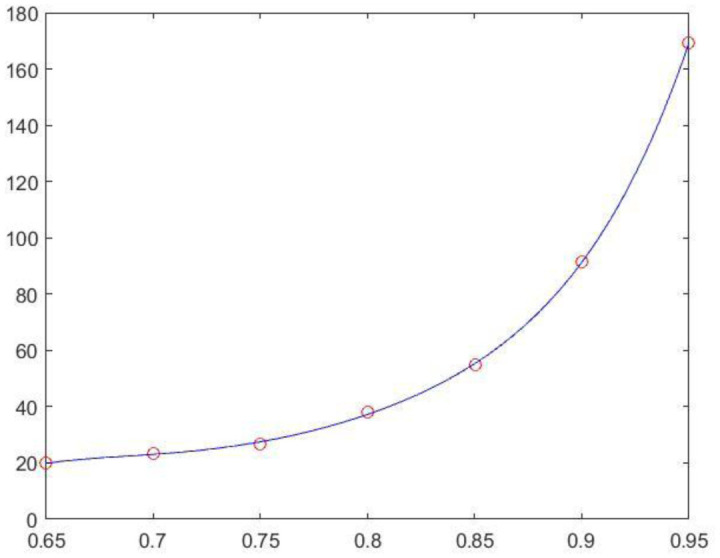
The relationship between gamma values and the partitioned module number.

The validity of MOSI was examined by t-tests for each subject (n = 33), hemisphere (n = 2) and gamma value (n = 7), and the *P* values were generally less than 10^−3^, and t-stats were greater than 4.08 (414 out of the 33*2*7 [=462] conditions had *P* values < 10^−3^). The results verify that MOSI reliably performs FP based on local homogeneity in terms of BOLD dynamics. It was discovered that the grand mean CCs increased with gamma values. The grand mean CC of the original partition informed by the Desikan-Killiany atlas was 0.33 (s.d. 0.034), which increased to 0.60 (s.d. 0.015) when gamma was 0.95. The resolution at gamma 0.65 was approximately 20 modules for each hemisphere, which increased to 170 modules when gamma reached 0.95; please see Table 1 and Figure 1 for details.

**Figure 2.**
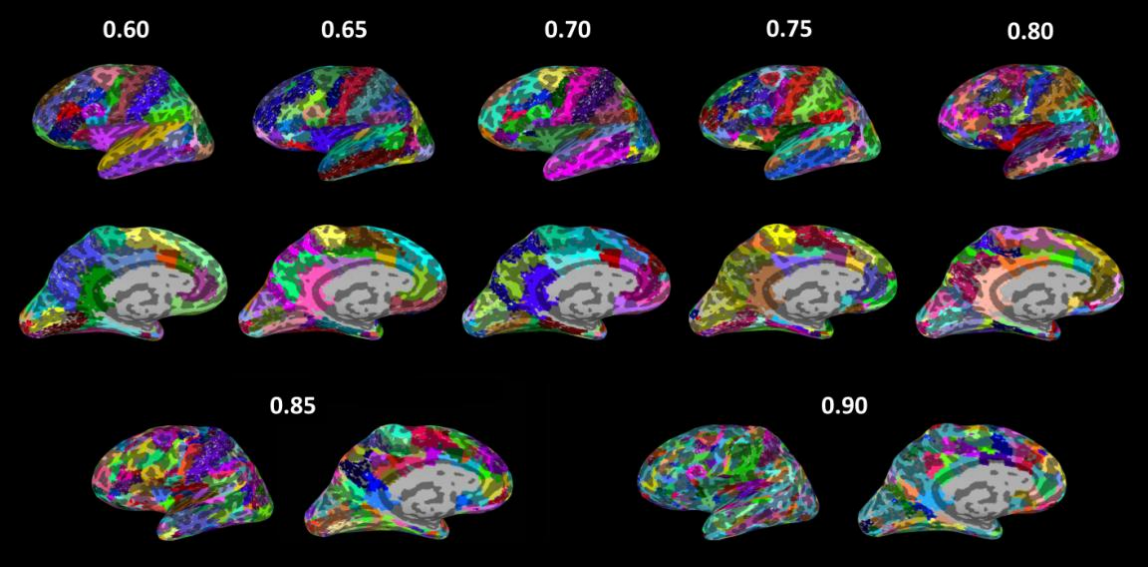
Example illustration of MOSI partitions derived from different gamma values (only the left hemisphere is shown). Each module is represented by a different color.

Paired t-tests of the grand mean CCs across all of the gamma values showed that the grand mean CC for a higher gamma value was always significantly greater than that for a lower gamma value, with most of the *P*-values of the pair-wise comparisons less than 10^−4^ (Bonferroni correction of *P-*value 0.05 is 0.05/28 pairs = 0.0018); please refer to Table 2 for details. For the above statistical comparisons, Fisher's transformation of CCs was also applied, and the conclusions remained the same (data thus not shown). It was found that MOSI worked efficiently, and the average calculation time was less than 6 minutes for gamma values no greater than 0.85 (Table 1; Memory 15.5 GiB, Processor Intel® Core™ i7-4700HQ CPU@2.40GHz, Disk capacity 1.0 TB). However, when gamma values were 0.90 and 0.95, the respective calculation time was approximately 8.5 and 26 minutes, indicating that the computation time of MOSI increased drastically when the cortex of a hemisphere was partitioned into greater than 90 communities (ROIs). A partition derived from different gamma values is illustrated using SUMA in Figure 2.

**Figure 3.**
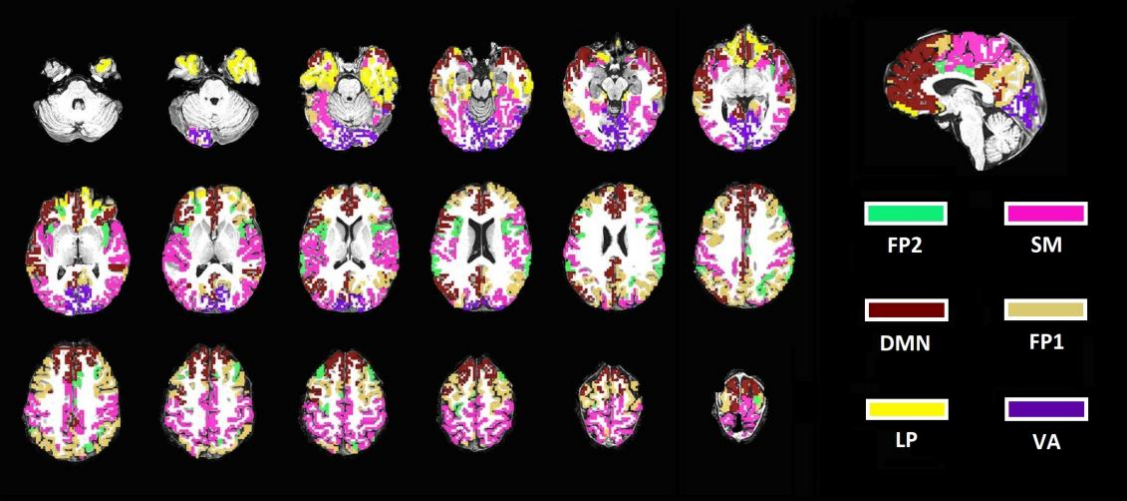
Example illustration of network components after the data-reduction procedure by MOSI (gamma = 0.8). Six networks are shown and labelled by colors: DMN, default-mode network; FP1, cognitive fronto-parietal network; FP2, attentional fronto-parietal network; LP, limbic-paralimbic area; SM, sensorimotor network; VA, visuo-associative network.

The MOSI results provide a novel start for data reduction of fMRI signals. Abridged (second level) FC maps were constructed accordingly (using the mean to represent all the voxels in a module and calculating the correlation matrix between the constituent modules). Another set of modular analyses were carried out consequently, revealing an interesting pattern. The authors attempted to identify the 5 commonly reported networks across the seven gamma values for each subject (i.e., visuo-associative, cognitive/attentional fronto-parietal, default-mode, sensorimotor and limbic/paralimbic networks); see an illustration by AFNI in Figure 3. There is inter-individual variation in terms of the spatial distribution of the constituent modules; however, the delineated modular structures are consistent and prominent, which is summarized in Table 3. With respect to conventional large-scale brain networks, the results suggest that a gamma value between 0.8 and 0.85 is reasonably good for individual analysis.

**Table 1.** Across different resolutions (7 gamma values) for the 15 subjects, grand means and standard deviations (s.d.) of correlation coefficients (CCs) were derived from the averaged CCs between the voxels in the constituent modules. Number is the mean modular number (left/right), and time is the summed calculation time of both hemispheres.

gamma	mean	s.d.	number (L/R)	time (sec)
original	0.33	0.034	25/25	n/a
0.65	0.40	0.036	20.2/19.5	124.0
0.70	0.41	0.040	23.2/23.4	199.2
0.75	0.41	0.036	26.9/26.9	317.8
0.80	0.43	0.026	38.6/37.6	337.7
0.85	0.47	0.022	55.3/54.2	359.5
0.90	0.53	0.018	91.7/91.4	511.7
0.95	0.60	0.015	169.3/169.3	1542.5

**Table 2.** Group-level paired t-tests of pair-wise comparisons of grand mean CCs across different gamma values (mean difference, upper triangle; t-statistics, lower triangle). Degree of freedom is 32.

gamma	original	0.65	0.70	0.75	0.80	0.85	0.90	0.95
original		0.07	0.07	0.08	0.09	0.13	0.19	0.26
0.65	12.8		0.01*	0.01*	0.03	0.06	0.14	0.21
0.70	15.6	2.1*		0.00*	0.02	0.06	0.12	0.19
0.75	17.4	2.8*	0.9*		0.02	0.05	0.11	0.17
0.80	25.0	6.8	5.3	5.9		0.04	0.10	0.13
0.85	31.4	13.7	12.3	14.5	14.8		0.06	0.07
0.90	34.9	23.8	20.0	21.3	25.3	18.9		
0.95	47.8	36.2	31.0	33.7	43.6	41.9	31.0	

Note: the sign of the number is ignored because the grand mean CC for higher gamma values is always higher than that of the lower gamma values. For all the pair-wise comparisons, the *P*-values are lower than 10^−4^, except the pairs *.

Network Scoring: 1, present; 0.5, present but defective (e.g., showing only one side of network A, and the other side of network A being confluent with network B, etc.); 0, absent. This arbitrary scoring system obeys two simple rules. First, the priority follows the order SM > FP > DM > VA > LP. For example, if SM is confluent with DM, then DM is 0, and SM is 1. If DM is divided into two parts and one part is united with SM, the score of DM would be 0.5. Integrity is the second consideration. For example, if FP is divided into several small parts, then its priority over DM, VA and LP is lost. However, if with increasing gamma, DM is divided into anterior and posterior components and neither is merged with other networks, then the score of DM is still 1.

Abbreviation: visuo-associative, VA; default-mode, DM; sensorimotor, SM; fronto-parietal executive, FP; limbic/paralimbic, LP.

**Table 3.** Identified networks by MOSI across 7 gamma values (expressed in percentage [%]).

gamma	VA	DM	SM	FP	LP
0.65	83	79	87	80	62
0.70	88	85	90	78	82
0.75	89	89	99	77	88
0.80	85	95	87	83	86
0.85	87	95	98	83	81
0.90	91	97	94	96	69
0.95	86	96	94	95	64

## 4. Discussion

It is believed that brain tissues that perform similar functions tend to aggregate together. Homogeneous cortical regions have been described using various tools, such as cytoarchitecture, white matter bundles, and anatomical landmarks. Given that the voxel number is large in fMRI (whole brain scanning may involve tens to hundreds of thousands of voxels) and the BOLD dynamics of voxels are not totally independent, it is justifiable to partition the cortex into communities with the content voxels sharing similar neural features. This process of dimensionality reduction will result in simplified neuro-informatics that are more feasible for further inferences about brain network properties. Given their theoretical and clinical importance, the research field of automatic FP of the cortex has been versatile, and various innovations have been used to segment the cortex automatically based on the functional characteristics of scanned voxels [28],[49]–[55]. Unlike previously proposed algorithms, MOSI utilizes the concordance in BOLD dynamics of the regional and neighboring voxels to parcellate the functional neuroimaging data. MOSI fulfills automatic segmentation of the cortex by combining modularity detection (dividing step) and similarity measurements (unifying step) that are repeated iteratively until convergence is reached. Using a Louvain community detection algorithm, the gamma value of the modularity formula may affect the resolution of the resultant parcellation [43],[44]. This feature is welcome because it is acknowledged that the central nervous system is organized at multiple spatial scales, ranging from neuron, tissue, gyrus, and area to lobe (or from column and map to system) [31],[56],[57]. In opposition to previous parcellation schemes that tended to yield fixed partition [32]–[35], MOSI may provide graded multi-scale solutions to cortical FP contingent upon research interest. The mean partition number for gamma values of 0.65–0.95 ranges from 40–340 (see Table 1). Intra-subject analyses consistently verified that the BOLD signals in each parcellated module by MOSI are more homogeneous than those in other modules. As expected, the homogeneity in terms of BOLD similarity within each module increases with higher gamma values (see Table 2).

A prerequisite for extracting reliable representative brain signals is to address the regional correlated BOLD signal structure. It is more comfortable to reduce the voxels with homogeneous content into a neural node and to use the mean time series to represent the brain signals embedded in those voxels. The data reduction achieved by MOSI, i.e., shrinking similar voxels to a node, provides a new starting point for subsequent network analysis. It is well known that neural activities in different brain regions are not independent from each other, which is also true for the BOLD dynamics of fMRI data. Investigation into distant neural synchronization has elucidated several large-scale networks in the brain [7],[39],[58], i.e., the brain has stable patterns of spontaneous activity that are organized as modular structures even in the resting state. After MOSI, a community detection algorithm was used again to unveil the hidden organization of the network structure, similar to many previous reports [6],[30],[45],[59]. It is noteworthy that under such a simplified scenario, community detection is used to categorize distantly linked neural nodes, which is in contrast to the analytic stream in MOSI where community detection is used to aggregate homogeneous and neighboring voxels. Regarding the 5 commonly reported large-scale networks, it was found that these network components can be identified at the individual level (except the attentional fronto-parietal network that may be confluent with the executive fronto-parietal network; see Tables 3 and Figure 3). Based on these results, we suggest that gamma values of 0.80–0.85 are suitable resolution for FP of rs-fMRI data, which corresponds to segmenting the cortex into 76–110 patches. Lower gamma values may help to study the interacting features between the networks listed above, while higher gamma values may help to unveil the neural features of the constituent sub-networks. Referring to the anatomical delineation of the limbic-paralimbic system [60], it is interesting to note that the derived limbic/paralimbic component occupied a much smaller region in previous reports and in this report, which was restricted to the orbitofrontal cortex and temporal polar region (some research did not even mention this component, see [30]) [28]. However, the limbic system (defined by anatomy/histology) and the midline/default-mode system (defined by rs-fMRI) overlapped substantially, nicely agreeing with the speculation of a recent study [39]. Despite the consistency, it is noteworthy that there is individual variability in terms of the spatial distribution of the constituent modules, and there are interesting individual-specific network patterns; for example, part of the precuneus may unite with the cuneus, the somatosensory module may correlate with anterior portion of the cuneus or part of the anterior cingulate cortex, the default-mode network may be divided into two sub-networks, insula or cingulo-insular network may be isolated, and the attentional fronto-parietal network may correlate with the cuneus (beyond the scope of this study, and the details thus not shown), etc. The variation might reflect individual neuropsychological characteristics that manifest at the large-scale network level, an area largely unexplored in current rs-fMRI research.

Given the importance of the FP topic in neuroimaging research, diverse analytic strategies have been developed and adopted to segment the cerebral cortex. It is appropriate to contrast our results with previous contributions to make the distinction of MOSI clear. There are three major categories of FP, briefly summarized below. The first class is the ROI-based approach that focuses on a particular structure. An ROI may be derived from the contrast of functional image analysis or, in an extreme case, may be reduced to a single voxel derived from the maximum activation of group-level analysis [39]. The second class is an atlas-based approach that pre-defines the cortex into tens to hundreds of regions and generally covers the whole brain [61],[62]. However, there has been a concern that the FP (and other measures, such as modularity analysis) based on atlas information may be inaccurate because the correspondence among different methods to define a homogeneous “functional” region is quite limited [10],[11]. If the validity of the upstream stage is questionable, the subsequent inferences about network properties could be biased (see alternative interpretation by [6]). There has been harsh criticism on the validity of atlas-based definitions of neural nodes [12],[13], which urges a data-driven approach to substantiate automatic whole-brain FP. The third class of FP is based on the features of brain signals per se, which provide a better representation of the brain dynamics. Various algorithms have been developed to serve the purpose of automatic whole-brain FP, such as sampling the brain via a regularly spaced grid and using whole-brain correlation information to guide FP or resorting to the trajectories of transition (abrupt changes in CC patterns based on each voxel) to define the parcellation boundaries [31],[34]. Expectation-maximization framework, variational Bayes approach, clustering techniques, independent component analysis and principal component analysis have all been incorporated into diverse FP algorithms under several experimental conditions [28],[49]–[55]. MOSI is different from all the above orientations in that local and neighboring homogeneity is the only pertinent consideration and constraint. Rather than extracting the “edge” that represents the boundary, MOSI respects the original definition of a module—the intensely connected members (voxels) are grouped together. After decomposing a larger module into several more homogeneous sub-modules, the neighboring sub-modules showing high similarity are united to form a new module. The splitting and unifying processes are repeated iteratively until the mutual information between the partitions of two successive cycles does not change. MOSI works fast, obeys the fundamental rationale to achieve data reduction (i.e., similar voxels are compressed to a node) and may lay the foundation for further individual network analysis. For example, the outputs of MOSI may provide novel starting points for multi-resolution structural network construction (e.g., the ROIs across different scales for diffusion tensor imaging analyses). The derived multi-resolution networks are innately multi-layered and may enable the exploration of the cross-scale network relationship. At each resolution, the output of MOSI may also endorse the study of scale-specific, multi-level network.

There are many clustering and community detection algorithms for FP of the brain but considering each of these algorithms is beyond the scope of this study. Nevertheless, it is well-known that different community detection algorithms may yield discrepant results (note: may also contribute to the individual variation in the network distribution described above) [6],[38],[63]–[65], which motivated Thirion et al. to examine the performance of different clustering algorithms for task-based fMRI datasets [66]. MOSI may provide a fair platform to enable the comparison of different community detection algorithms and to select the optimal algorithm/parameters tailored for resting-state brain network description. Other potential applications of MOSI include investigating the issue of hemispheric asymmetry [67], clarifying the debate on structural and functional concordance [45], characterizing network/graph properties, etc. It is also of interest to apply MOSI in neuropsychiatric populations and in the study of neural reorganization/plasticity. The authors expect that using MOSI as a data-reduction (pre-processing) procedure may enhance the consistency and comparability of network research in the field of rs-fMRI research.

## 5. Conclusions

A major challenge for FP of whole-brain fMRI data lies in the large voxel number; therefore, data-reduction strategies are frequently applied, such as regularly spaced seeds and atlas-defined ROIs [4],[34]. In contrast to previously proposed algorithms [31],[34], MOSI utilized the concordance in BOLD dynamics of the regional and neighboring voxels to parcellate the functional neuroimaging data and to achieve data reduction. By titrating gamma values, MOSI may also adapt the partition to different resolutions. The validity was confirmed by intra- and inter-individual analyses. The empirical analysis suggested that gamma values of 0.80–0.85 seem to be suitable for FP of the cortex to commonly reported networks. MOSI may provide a novel starting point for subsequent network analysis of rs-fMRI data.
